# Treatment Delay and Total Delay among Pulmonary Tuberculosis Patients in the North of Iran: Application Survival Data Analysis

**Published:** 2017

**Authors:** Jamshid Yazdani-Charati, Mohammad Sadegh Rezai, Afsane Fendereski, Soraya Mohammadi, Nadia Alipour

**Affiliations:** 1 Department of Biostatistics, Health Sciences Research Center, School of Health Sciences, Mazandaran University of Medical Sciences, Sari, Iran; 2 Infectious Disease Research Center with Focus on Nosocomial Infections, Mazandaran University of Medical Sciences, Sari, Iran; 3 Students Research Committee, Department of Biostatistics, School of Health, Mazandaran University of Medical Sciences, Sari, Iran.

**Keywords:** Tuberculosis, Cox Regression, Survival, Treatment Delay, Total Delay

## Abstract

**Background:**

Tuberculosis (TB) remains the leading cause of death among infectious diseases worldwide. Identifying the factors associated with the treatment delay and total delay would be helpful in the prevention of tuberculosis and in reducing the burden on the health care system. The objective of this study was to assess the treatment delay and total delay in TB patients and investigate the factors causing these delays.

**Materials and Methods:**

This was a longitudinal study conducted in 2009–2015. Our study consisted of 1694 TB patients registered in the TB center of Mazandaran province. Data regarding the patients’ demographic characteristics and clinical factors associated with treatment delay and total delay were analyzed. Kaplan Meier plots and log rank tests were used to assess the survival pattern. Cox proportional hazards model for multivariable analysis was discussed. We used mean values and median (Q2) [first quartile (Q1)-third quartile (Q3)] to describe delays.

**Results:**

The median treatment delay and total delay were 35 (ranged 23–80) and 36 (ranged 24–82) days, respectively. The mean age of TB patients was 47.40±20.3. No significant association was found between the location of residence, nationality, gender, and type of pulmonary TB patients with treatment delay and total delay. Additionally, age, prison status of patients, HIV test, and contact history had a significant relationship with the treatment delay and total delay (p-value <0.05). It was shown that the median total delay in men patients in the ≤14 year-old age group, imprisoner patients, rural patients, patients who have not received an HIV test, smear negative patients, those who are Iranian, and TB patients whose contact history was unknown was lower than that of others. The highest median treatment delay and total delay was in the >60 age groups, and were 41 and 44 days, respectively. Treatment delay was the same as the total delay except in the place of residence variable; median treatment delay among urban patients was less than that of rural patients.

**Conclusion:**

According to this study age, prison status of patients, HIV test and contact history had a significant relationship with the treatment delay and total delay (P-value<0.05). Understanding the factors that are closely associated with these delays is essential to effectively control TB and could be helpful in reducing these delays.

## INTRODUCTION

Despite the existence of effective treatment methods, tuberculosis (TB) remains one of the major infectious diseases prevalent in most countries (1–3). Based on a WHO report, there were an estimated 10.4 million incident TB cases worldwide, of which 5.9 million (56%) were among men, 3.5 million (34%) among women and 1.0 million (10%) among children (4). The incidence rate in Iran was reported as 12.6 per 100,000 population, and this rate is the highest in Sistan-Baluchistan and Golestan provinces (5, 6).

A national survey conducted in Iran in 2003 showed that the median delay in diagnosis and treatment in patients with sputum positive pulmonary tuberculosis was 92 days (with an average of 120+10 days) and the median patient delay and median physicians delay in diagnosis was 20 (with an average of 44+6 days) and 46 days (with an average of 76+8 days), respectively (7). Transmission of TB is difficult to control, and one index case may infect a large number of secondary cases if left untreated (8–10); therefore, reducing the time delay from symptom onset initiation of treatment is required for better management of TB (9,11). The delay in treatment could be due to the delay in diagnosis by the physicians or due to the failure of the treatment provided, and due to these reasons, treatment delay may increase (12). Delay in accessing TB care is common in both industrialized and developing countries. However, there is no international consensus on what constitutes an acceptable delay (13). The average delay in low-income countries has been reported to be 9.7 weeks (14). Since delays may increase mortality, the factors affecting treatment delay are of key importance in TB management (14, 15). Since these delays may have adverse effects on the wellbeing of the society and the health system by possibly increasing the treatment duration, the aim of this study was to evaluate the survival of these patients and determine the factors that cause treatment delays in Mazandaran. Our study results would be useful for the health authorities and strategic planners in the design and implementation of interventional TB control programs, and in finding ways to treat and diagnose patients as early as possible.

## MATERIALS AND METHODS

### Study participants or Data collection

This was a longitudinal study conducted during 2009–2015 in Mazandaran province. The names and contact information of all patients presented to the medical centers affiliated to Mazandaran University of Medical Sciences and Babol University of Medical Sciences were extracted from the TB registry. This data extraction was performed in compliance with the rules and regulations of the ethics committees of Mazandaran University of Medical Sciences and Babol University of Medical Sciences. Patients suspected of having TB based on signs and symptoms were diagnosed using the sputum smear test and pathological examinations by the physicians of the health centers or private clinics, and were then referred to TB registry centers for medical treatment. Patients who their first symptom date or diagnosis date or treatment dates were unknown considered as censored data. All the data regarding 1694 TB patients who had various forms of TB were collected from 18 areas in Mazandaran province during the 6-year period. Demographic characteristics, such as age, gender, type of disease, HIV testing (whether patient has been tested for HIV or not), nationality, prison status (patients who were in prison or not), contact history, date of diagnosis, date of first symptom(s), date of treatment, and residential location were evaluated.

### Data analysis

We assessed the demographic characteristics affecting the treatment delay and total delay in patients, using Cox semi-parametric regression analysis to determine the relationship with the probability of survival in all subgroups of the population relative to baseline category (BL), and to determine the hazard ratio. T being the treatment delay or total delay (days), the value of T can reflect a probability distribution. The main form of this model consists of:

$$\text{ln}(h(T))=\text{ln}(h_{0}(T))+\sum_{i=l}^{p}|x_{0}\beta_{i}$$

In this model, *x*_1_, *x*_2_,…,*x_p_* are covariates, *β*_1_,*β*_2_,…, *β_p_* are estimated regression coefficients, and *h*_0_(*T*) is the baseline hazard rate when all covariates are equal to zero (16). First, we used the Kaplan-Meier plot for treatment delay (time between diagnosis and the treatment date) and total delay (time between the first symptom(s) and the treatment time); then we compared this using log-rank test. Log rank (Mantel–Cox) test for the equality of survival distributions was used to analyze the significance of survival difference among the categorical variables. For single variable analysis p<0.4 was considered significant. We then performed Cox proportional hazards regression analysis to evaluate the impact of age, gender, nationality, place of residence, prison status (patients who were in prison or not), date of diagnosis, date of the first symptom, date of treatment, contact history with TB patient (we defined dummy variable for contact history), Type of pulmonary TB (Smear positive, Smear negative), HIV test status, treatment delay. We used mean and quartiles median (Q2) [first quartile (Q1) - third quartile (Q3)] to describe delays. Statistical analyses were performed using SPSS Ver. 16 software; and the value of α=0.05 was considered significant.

## RESULTS

Of the 1694 registered TB patients selected for analysis, 1120 (48.2%) were male patients and 574 (24.7%) were female patients. Additional information is shown in Table 1. The mean age of male and female TB patients was 45.97±19.71 and 50.19±22.35 years, respectively. The mean age of all patients was 47.40±20.3. The median (Q1- Q3) treatment delay and total delay were 35 (23–80) and 36 (24–82) days, respectively. Moreover, the mean treatment delay and total delay were 68.29 and 69.9 days, respectively. Table 2 illustrates the Cox regression analysis. First, variables with p-values less than 0.4 in log rank test were entered in the Cox regression model. After distinguishing those variables, we used the semi-parametric Cox and determined the variables affecting the treatment delay and total delay.

**Table 1.** Characteristics of study participants (N=1694) in Mazandaran, Iran, 2009–2015

**Characteristic**		**N(percent)**	**Treatment delay**				**Total delay**			

			**Mean**	**Median**	**Quartiles**		**Mean**	**Median**	**Quartiles**	
					**1st**	**3rd**			**1st**	**3rd**
**Gender**	Female	574(33.88)	71.47	38	23	80	73.16	40	24	82
	Male	1120(66.12)	66.65	33	22	79.25	68.26	35	24	82

**Age group**	<14	17(0.7)	39.64	28	11.5	38	40.35	28	11.5	38.5
	15–24	172(7.4)	50.23	31	20	64.5	51.65	31	23	66.25
	25–39	558(24)	63.33	33	23	74	64.30	36	24	74.5
	40–60	439(18.9)	71.89	34	20	87.25	74.71	36	22	90
	>60	508(21.8)	77.61	41	24	90	79.05	44	26	90

**Prison status**	Yes	192 (8.3)	45.06	30	18	31	46.72	30	19	33
	No	1502(64.6)	71.24	38	23	84.25	72.87	41	25	88

**Residence**	Rural	687(40.5)	67.33	35	24	78.5	68.83	36	25	80
	Urban	1007(59.45)	68.93	34	22	80	70.65	37	23	82

**HIV test status**	Known	301(17.77)	75.22	57	31	91	77.15	59	31	92
	Unknown	1393(82.23)	66.79	31	20.75	73	68.36	33	22	76

**Type of pulmonary TB**	Smear positive	1324(56.9)	67.96	36	25	81	69.7	38	27	84
	Smear negative	370(15.9)	69.4	32	15	70.25	70.67	33	16	72.25

**Nationality**	Iranian	1653(71.1)	67.98	34	22.25	79.75	69.6	36	24	81.75
	Afghan	41(1.8)	80.43	53	28.5	95	82.68	55	30	95

**Contact History**	Yes	275(11.8)	70.96	42	25	92	72.63	45	26	92
	No	702(30.2)	83.39	54	30	92	85.53	56	30	93
	Unknown	715(30.7)	52.18	30	15	36	53.32	30	16	59

**Table 2.** The significant variables in the Cox multi-variable for the patient’s with TB in Mazandaran province during 2009–2015

**Variable**		**Treatment Delay**			**Total Delay**		

		HR^*^	Hazard ratio (95% CI)	P-value	HR	Hazard ratio (95% CI)	P-value
**Age**		0.996	0.994–0.999	0.007	0.997	0.994–0.999	0.008

**Gender**	Female(BL)						
	Male	0.971	0.874–1.080	0.592	0.975	0.877–1.084	0.635

**Type of pulmonary TB**	Smear positive(BL)						
	Smear negative	1.105	0.983–1.243	0.095	1.116	0.992–1.255	0.068

**Prison status**	Yes(BL)						
	No	1.351	1.149–1.590	<0.001	0.722	0.614–0.849	<0.001

**Nationality**	Iranian (BL)						
	Afghan	0.814	0.594–1.115	0.201	0.812	0.593–1.113	0.196

**HIV test status**	Known(BL)						
	Unknown	1.287	1.132–1.463	<0.001	1.286	1.131–1.463	<0.001

**Contact history**	No(BL)						
	Yes	0.929	0.806–1.071	0.309	0.929	0.806–1.071	0.309
	Unknown	0.675	0.605–0.753	<0.001	0.668	0.599–0.746	<0.001

* HR: Hazard ratio

Table 2 shows that the variables of age, prison status of patients, HIV test, and patients who their contact history were unknown had significant relationship with treatment delay and total delay (p-value<0.05). As the p-value for age was less than 0.05, there were differences in total delay and treatment delay for age variable. With respect to hazard ratio for age variable, we noted that each 1-year increase in age resulted in almost 99.6 % decrease in both delays.

A significant difference was observed between total delay and treatment delay for patients who their contact history were unknown (p-value≤0.001). The longest mean (median) estimated times for total delay was 85.53 (56) days and 83.39 (54) days for treatment delay. The risk of increase in treatment delay and total delay for patients who their contact history’s status with another TB patient were unknown was 67.5% and 66.8% less than patients whose had not contact history (baseline category), respectively.

As the p-value was <0.001 for both delays with respect to prison status, we concluded that there was significant evidence for the difference in survival times. The longest mean (median) estimated time for total delay was 72.87(41) days, for patients that were not in prison and the shortest total delay was 46.72(30) days, for prisoner patients. Hazard ratios were 1.351 and 0.722 for treatment delay and total delay, respectively. Therefore, treatment delay for patients who are not in prison was 35.1 times more than that of prisoner patients and total delay for patients who are not in prison was 72.2 percent less than that of prisoner patients.

Since the p-value variable for both delays was less than 0.05 with respect to the HIV testing, we concluded that there was significant evidence for a difference in survival times for patients who received HIV test regardless of the test result. It was noted that both delays were 28.7 % times greater in patients who did not receive an HIV test as compared to those who received an HIV test.

**Figure 1.**
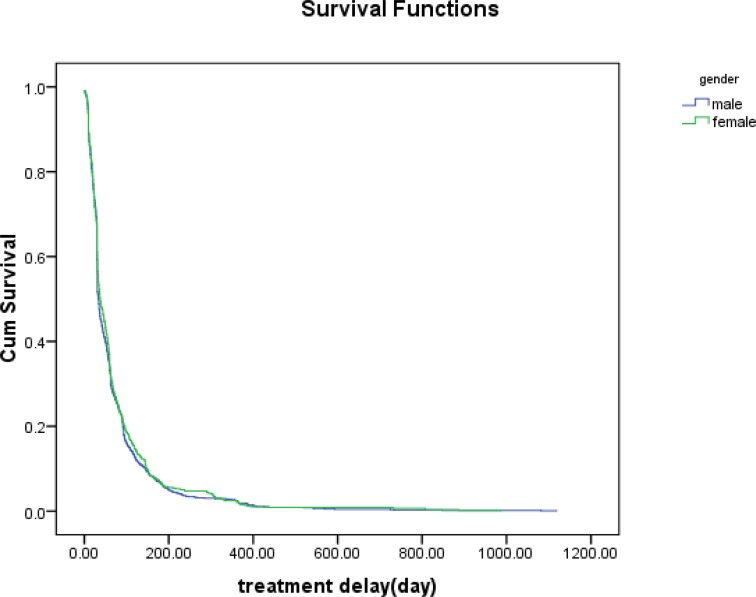
The survival function for the gender with the TB disease against time (treatment delay) in Mazandaran province from 2009 to 2015.

**Figure 2.**
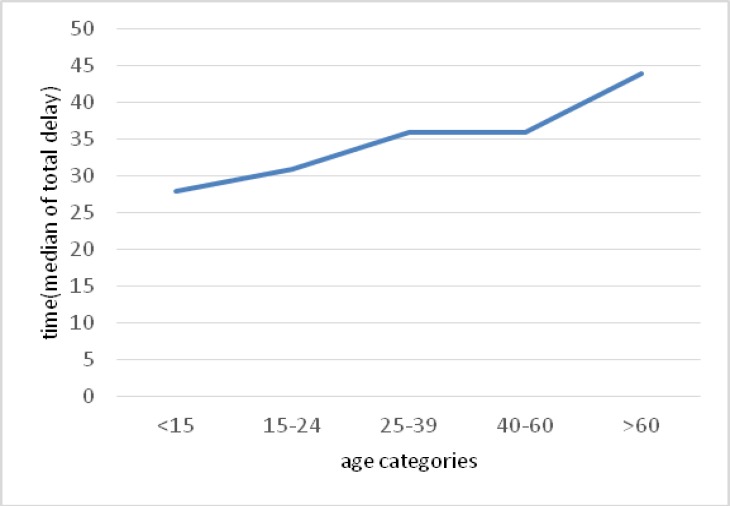
The median total delay by age categories with the TB disease in Mazandaran province from 2009 to 2015.

The estimated median (mean) values for the treatment delay was 33(66.65) days for male patients and 38 (71.47) days for female patients. In addition, the estimated median (mean) of total delay was 35 (68.25) days for male patients and 40 (73.16) days for female patients. There were no differences in total delay and treatment delay for male patients and female patients (Figures 1 and 2). The median (mean) time between the first symptom(s) date and treatment date (total delay) was 36 (69.60) and 55 (82.68) for Iranians and Afghans, respectively. However, there were no differences in total delay and treatment delay between Iranians and Afghans. Although there were no differences between smear positive patients and smear negative patients, both treatment delay and total delay for the smear positive patients were greater than that of the smear negative patients.

## DISCUSSION

This study was designed to explore the treatment delay and total delay among TB patients and to explore relationships between various characteristics of the study participants and patients’ treatment delay and total delay in Mazandaran province in 2009–2015. In our study, the median treatment delay was found as 35 while total delay was 36 days. We have found that the median total delay was lower in men, patients in the ≤14 year-old age group, prisoner patients, rural patients, patients who have not received an HIV test, smear negative patients, those who are Iranian, and patients whose contact history was unknown. Treatment delay was the same as total delay, except for the variable of place of residence; the median treatment delay among urban patients was less than that among rural patients.

A systematic review that investigated delays in diagnosis and treatment of pulmonary tuberculosis in India revealed that the median treatment delay was 2.5 days (1.9–3.6) which was shorter than the treatment delay found in our study (17). Other studies have also found shorter median for treatment delay, such as those in Guinea-Bissau (12.1 weeks) (18), in Tanzania (28 days) (19), in Botswana (5 weeks) (20), in Ethiopia (2 days) (21), and in Nigeria (11 weeks) (22). In a study conducted in Nigeria, it was found that the median treatment delay was one week and the median total delay was 10 weeks, which were longer than the delays found in our study (23).

Another study (24) which investigated the diagnostic and treatment delays for tuberculosis in 7 countries within the eastern Mediterranean region revealed that the longest mean of treatment delay was in Iran, with 127 days; however, our study showed a lower delay of 68.29 days.

In a study conducted in Markazi province, the results showed that the mean of total delay and treatment delay were129.9±171 and 2.1±5.2, respectively. In addition, there were no significant associations between gender and nationality with treatment delay (25). However, the mean of total delay and treatment delay were found to be 69.93 and 68.29 in our study, both of which are greater than the abovementioned study. On the other hand, there were no associations between gender and nationality with treatment delay in both studies. The differences between values found for delays could be due to variation in the selection of the study population, difference in treatment delay definition as they may differ in various countries. Our findings could be related to poorer transportation system in Mazandaran province.

In a study by Lorent et al., one of the risk factors for delay in the diagnosis and treatment of TB among 104 patients with a mean age of 35 years (range 17–84) was the patient’s HIV status as 62% was found to be HIV-positive; no significant association was found between treatment delay, age, and gender (26). However, our results showed significant association between patients who received an HIV test and those who did not, and treatment delay and total delay.

This might be due to the difference in the number of patients, as there were 104 patients in the study by Lorent, N et al. while our study had 1694 patients. Additionally, the number of patients who were HIV-positive is significantly higher in that study (26 HIV-positive patients) as compared to our study. The possible reason for this difference might be that the patients in our study may not have had the inclination to take an HIV test; therefore, the HIV status of most of the patients in our study was unknown.

In a study by Charati and Kazemnegad, 89.2% of TB patients identified in Mazandaran province in 2008 were not evaluated for HIV. Only 25 TB cases were examined for HIV and 3 of those were HIV positive. In this study, the percentage of patients whose HIV status were unknown was 82.2%, but the number of patients who received the test were 301 (17.77%) cases which shows that HIV testing had increased since the above mentioned study (5).

Chiang, et al. (27) studied the patient- and health system-related delays in the diagnosis and treatment of tuberculosis in a population-based patient interview study. Results showed that being younger than 65 years was associated with longer treatment delay; based on the findings from many other studies, diagnosis and treatment delays were found to significantly increase with age (28–35).

In our study, there were significant associations between age and treatment delay, with the median treatment delay and total delay increasing with the age. It might be due to the feeble performance of the physicians in the diagnosis of TB among elderly people. This weakness in the diagnosis may be due to the possibility that physicians are not able to recognize and differentiate TB symptoms when there are underlying diseases. Information on better case detection should be included in trainings to familiarize the personnel and medical staff with the signs and symptoms of TB; equipping the laboratories appropriately will also enable them to diagnose TB cases better and efficiently (17).

In a study by Lienhardt et al., a total of 152 TB patients were interviewed. The median delay from the onset of symptoms to commencement of treatment was 8.6 weeks (range 5–17). Treatment delay was independent of gender, but was shorter in young TB patients. The median delay was longer in the rural areas than that in urban areas (28). In our study, it was found that total delay was less than the abovementioned study and women had longer treatment delay than men, which may be because many women depend on their husbands or male relatives to bring them in for treatment because of the difficulties with transportation (36).

The high number of TB patients found in the rural areas 687 (40.5%) indicate the availability of more efficient health care service and better economic status in the rural areas as compared to the cities and urban areas. Our result in this regard was in agreement with a study that was conducted by Yazdani-Chrati J et al. (37).

According to the results the high percent of patient’s contact history were unknown (30.7%) and had significant relationship with total and treatment delays. The hazard ratio for patients who their contact history’s status was unknown was less than patients who had not contact with TB patients. It can due to patients who their contact history were unknown were belonged to patients who had contact history category and didn’t evaluate and assess completely.

As our results showed the longest estimated median time for treatment delay and total delay were among patients who are not in prison versus patients who are in prison and were 38 and 41 days, respectively.

In addition, 2.1% of the 192 prisoners who received an HIV test were infected with HIV. This suggests that if all prisoners were to receive the HIV test, the number of diagnosed cases would increase. Moosazadeh et al. published a short report on the incidence of TB among the prisoners in Mazandaran province, showing that 7 prisoners (0.3%) were infected with HIV, and overall 147 (6.1%) of the prisoners were TB patients (38).

## CONCLUSION

The estimated median treatment delay and total delay were 35 and 36 days, respectively. We also found in our study that variables such as age, prison status, HIV testing, and patients who their contact history were unknown, had a significant relationship with the treatment delay and total delay (p-value<0.05).

Considering that the delay in TB treatment in our study was high and that TB is a contagious disease, it is recommended that diagnostic methods for TB should be emphasized in the training courses for medical students, continuing education courses for general physicians and specialists, and in post-graduate courses. To reduce the treatment delay, clinics need to be more involved and the referral mechanism must be strengthened. We also need to improve awareness of the symptoms of tuberculosis both in the general public and among health care professionals. There has not been any studies regarding the delays among pulmonary tuberculosis patients in Mazandaran province and this study reports the situation regarding the delays among pulmonary tuberculosis patients in Mazandaran province, and provides recommendations for improvements to help with the planning to reduce these delays, and hence reduce the period of treatment. One of the limitations of our study was that the registry data had recording errors. Additionally, it is possible that not all of the patients in this province had been registered. We also noted that the information on social and economic variables was not complete and these variables could have an effect on treatment delay and total delay.
